# Integrated analysis of the responses of a circRNA-miRNA-mRNA ceRNA network to heat stress in rainbow trout (*Oncorhynchus mykiss*) liver

**DOI:** 10.1186/s12864-020-07335-x

**Published:** 2021-01-11

**Authors:** Jinqiang Quan, Yujun Kang, Zhicheng Luo, Guiyan Zhao, Lanlan Li, Zhe Liu

**Affiliations:** College of Animal Science & Technology, Gansu Agricultural University, Lanzhou, 730070 P.R. China

**Keywords:** Rainbow trout, Heat stress, Transcriptome, ceRNA network

## Abstract

**Background:**

With the intensification of global warming, rainbow trout (*Oncorhynchus mykiss*) suffer from varying degrees of thermal stimulation, leads to mass mortality, which severely restrict the development of aquaculture. Understanding the molecular regulatory mechanisms of rainbow trout under heat stress is useful to develop approaches to relieve symptoms.

**Results:**

Changes in nonspecific immune parameters revealed that a strong stress response was caused in rainbow trout at 24 °C, so we performed multiple transcriptomic analyses of rainbow trout liver under heat stress (HS, 24 °C) and control conditions (CG, 18 °C). A total of 324 DEcircRNAs, 105 DEmiRNAs, and 1885 DEmRNAs were identified. A ceRNA regulatory network was constructed and a total of 301 circRNA-miRNA and 51 miRNA-mRNA negative correlation pairs were screened, and three regulatory correlation pairs were predicted: *novel_circ_003889* - *novel-m0674-3p* - *hsp90ab1*, *novel_circ_002325* - *miR-18-y* - *HSPA13* and *novel_circ_002446* - *novel-m0556-3p* - *hsp70*. Some target genes involved in metabolic processes, biological regulation or response to stimulus were highly induced at high temperatures. Several important pathways involved in heat stress were characterized, such as protein processing in the ER, the estrogen signaling pathway, and the HIF-1 signaling pathway.

**Conclusions:**

These results extend our understanding of the molecular mechanisms of the heat stress response and provide novel insight for the development of strategies that relieve heat stress.

**Supplementary Information:**

The online version contains supplementary material available at 10.1186/s12864-020-07335-x.

## Background

Rainbow trout (*Oncorhynchus mykiss*) is a typical cold-water fish [1] and is one of the excellent breeding species recommended by the FAO worldwide (Food and Agriculture Organization of the United Nations). Because of fast growth and high adaptability, rainbow trout has become the highest-yielding species of freshwater fish in China. With the intensification of global warming, high temperatures severely restrict the development of rainbow trout aquaculture in the summer. Animals undergo stress in response to a variety of conditions, including transient exposure to hot or cold temperatures, heavy metals, hypoxia stress, etc. [2, 3]. When rainbow trout are subjected to heat stress, the physiological functions of these animals are disturbed; for instance, the balance of the oxidation-antioxidant systems is disrupted. Simultaneously, heat stress causes oxidative damage in cells, reduces the immunity of organisms, and even leads to death under severe conditions [4]. However, the organism will initiate a stress defense response due to adaptive regulation under conditions of heat stress [5]. Previous studies have shown that the adaptive regulation of organisms in response to heat stress was mainly in the differences in transcription levels [6–8], and was also accompanied by the regulatory effect of noncoding RNA on protein metabolism, immune response, apoptosis, etc. [9].

Circular RNAs (circRNAs) are covalently closed, endogenous noncoding RNAs that are involved in many cellular and developmental processes in eukaryotic cells [10–13]. CircRNAs possess the significant characteristic of noncanonical splicing without a free 3′ or 5′ end; thus, they cannot be degraded by ribonuclease and are highly stable [14]. CircRNAs have tissue- and developmental stage-specific expression patterns, owing to their abundance, stability and diverse expression profiles, and likely play a pivotal role in various biological activities and regulatory pathways; for instance, a few studies revealed that circRNAs could serve as miRNA sponges and thereby impair miRNA-mediated gene silencing [11, 15]. Moreover, circRNAs may bind to transcription factors (TFs) and RNA-binding proteins (RBPs), forming ribonucleoprotein complexes with specific functions [16, 17], or they may act as protein decoys or antagonists, modifying the cellular destination and/or function of bound factors, such as circ-Foxo3 [18].

Recent research has shown that mRNA is not a unique target for miRNA regulation, and noncoding genes also play an important role in miRNA-mediated expression regulation [19, 20]. RNAs with the same miRNA response element (MRE) are able to compete for binding to miRNAs, which are called competitive endogenous RNAs (ceRNAs). ceRNA is a new mechanism of interaction between RNAs [21, 22]. ceRNA can be used as a bait to attract and isolate miRNAs, further lifting the inhibition of the target gene by the miRNA. Communication through newly discovered MREs allows mRNA, circRNA and pseudogenes to achieve mutual regulation through miRNA competition mechanisms, representing a large-scale posttranscriptional regulatory ceRNA network [21, 23–25]. As miRNA sponges, circRNAs could negatively regulate the activity of miRNAs through the ceRNA network, further regulating the expression of mRNA. However, the regulatory mechanism of ceRNA in rainbow trout under heat stress remains unclear. In this study, the circRNA, miRNA and mRNA of rainbow trout under heat stress were mined by high-throughput sequencing and bioinformatics analysis. The identification and characterization of the regulatory mechanism of ceRNA is intended to provide improved recommendations for relieving heat stress in rainbow trout.

## Results

### Effects of heat stress on nonspecific immunity in rainbow trout

**Fig. 1:**
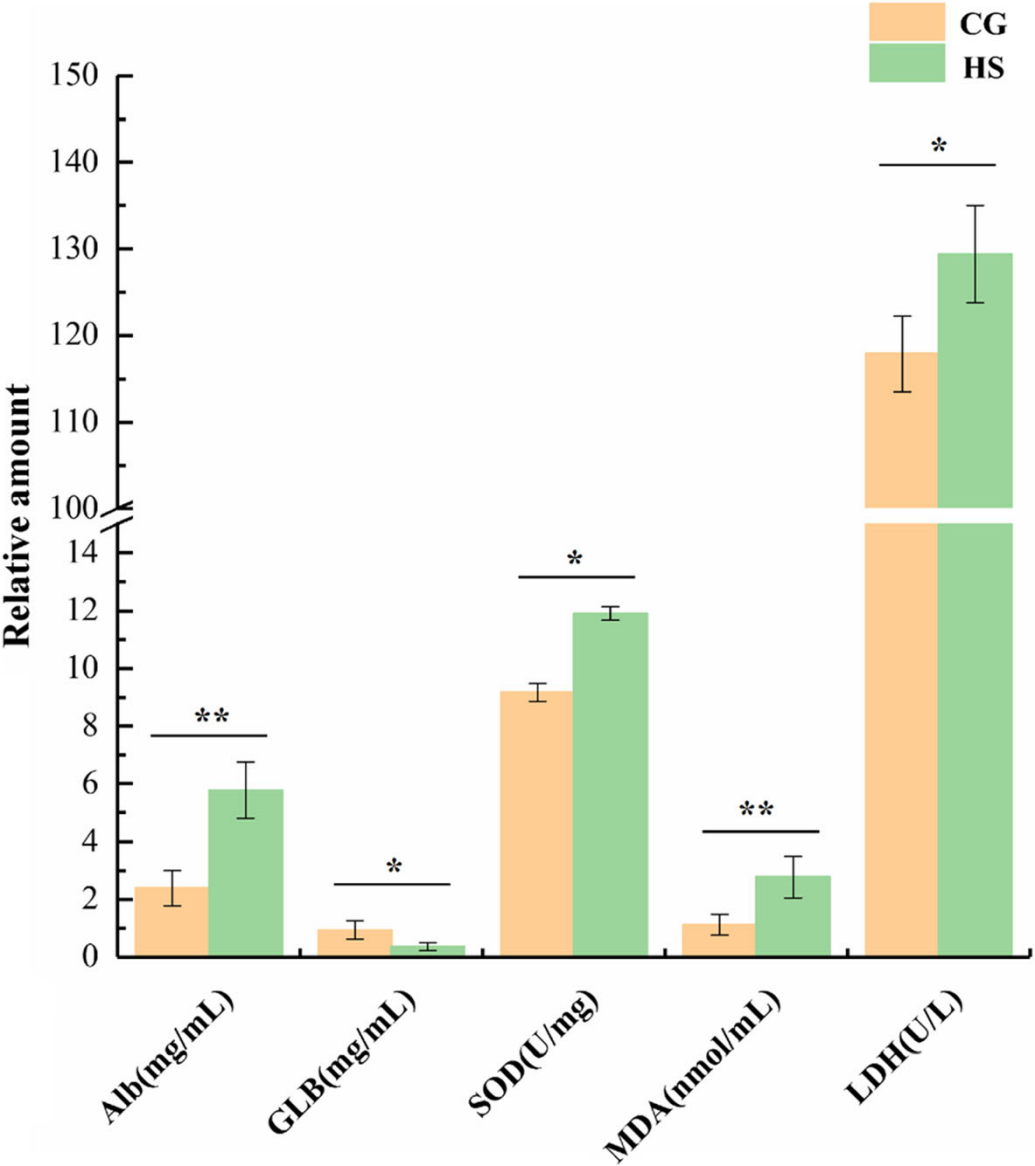
Change in nonspecific immunity in rainbow trout under heat stress

Superoxide dismutase (SOD) activity and lactate dehydrogenase (LDH) activity in the liver tissue of rainbow trout under heat stress (24 °C) were significantly increased (*P*< 0.05; Fig. 1), albumin (Alb) content and malondialdehyde (MDA) content were highly significantly increased (*P*< 0.01), but globulin (GLB) content was significantly decreased (*P*< 0.05).

### Overview of RNA-seq results in rainbow trout under heat stress

Six cDNA libraries, including three from the control group (CG: CG-1, CG-2, and CG-3) and three from the heat stress group (HS: HS-1, HS-2, and HS-3), were constructed and analyzed by high-throughput sequencing. A total of 100.3 G clean bases were obtained and deposited in the National Center for Biotechnology Information database under the accession number GSE135668 (circRNA and mRNA) and GSE140112(miRNA). After quality control and filtering the raw reads, 658,927,256 high quality clean reads were generated from the six libraries. Then, the comparison software Bowtie2 (2.2.8) was used to compare high quality clean reads to the ribosome of the species (mismatch number: 0) and to remove the reads corresponding to ribosomal RNA, and 658,860,062 effective reads were obtained. The average Q30 was 94.83%, and the average GC content was 50.93%. The ribosomal RNA reads were filtered based on the updated reference genome of rainbow trout [26], and the majority of effective reads were successfully mapped; the average of mapped ratio was 76.90%. The mapped reads represented slightly more than 75% of the rainbow trout genome; therefore, the differentially expressed genes (DEGs) analysis based on the genome was reliable.

### Identification of DEcircRNA, DEmiRNA and DEmRNA in rainbow trout liver tissues under heat stress

**Fig. 2:**
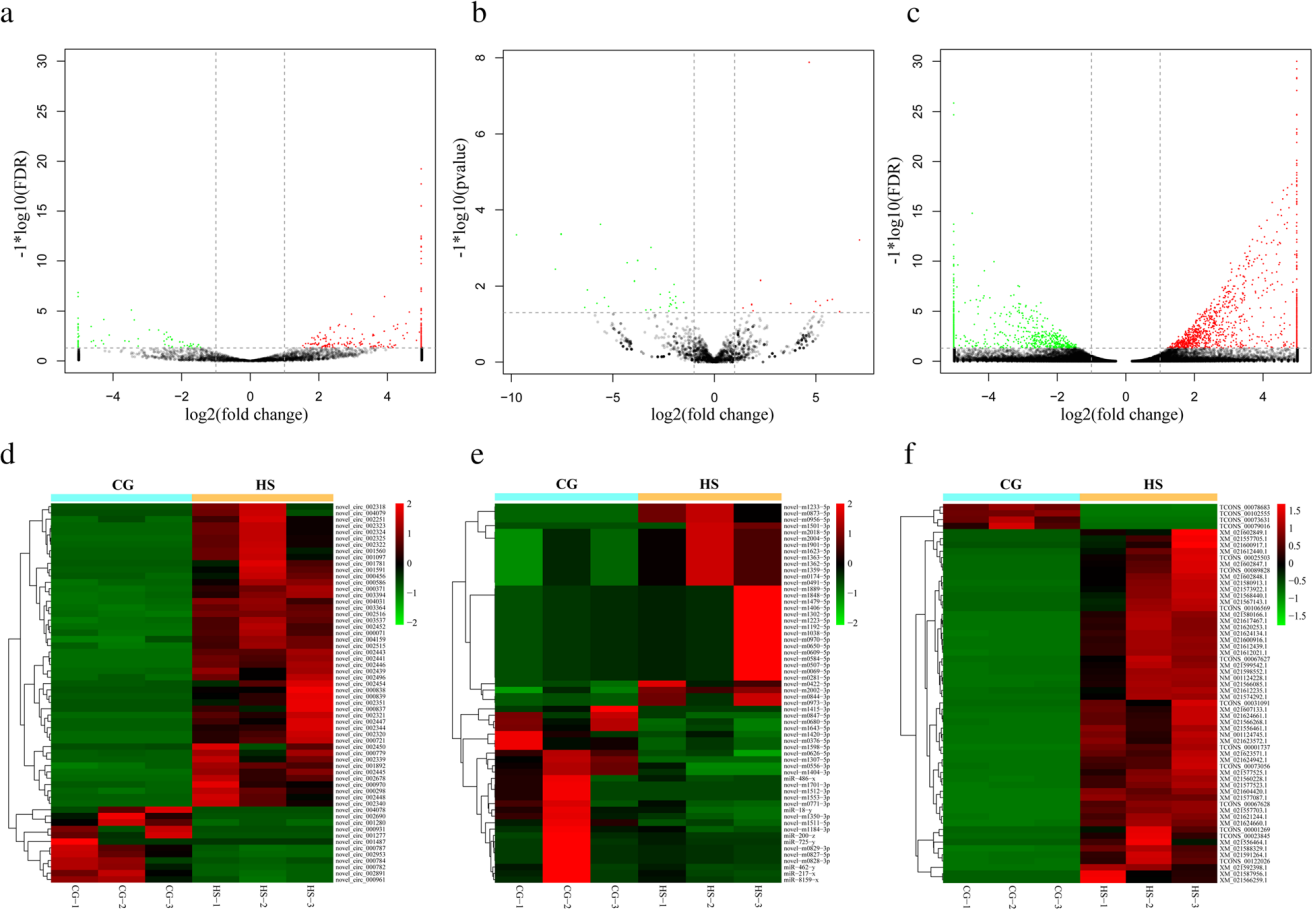
**a** Volcano plot of DEcircRNAs. **b** Volcano plot of DEmiRNAs. **c** Volcano plot of DEmRNAs. **d** Heat map of the top 60 DEcircRNAs. **e** Heat map of the top 60 DEmiRNAs. **f** Heat map of the top 60 DEmRNAs

A total of 4138 circRNAs were obtained from the six libraries, and 324 significantly differentially expressed circRNAs (DEcircRNA) were identified by false discovery rate (FDR) < 0.05, among which 247 DEcircRNAs were upregulated, and 77 DEcircRNAs were downregulated (Fig. 2a and Supplementary Table S1). The top 60 DEcircRNAs are presented in a heat map based on gene expression (Fig. 2d). Additionally, a total of 2730 miRNAs and 67,107 mRNAs were obtained, 105 significantly differentially expressed miRNAs (DEmiRNA) were identified by stringent thresholds (FDR< 0.05), among which 51 differentially expressed mRNAs (DEmRNA) were upregulated, and 54 DEmiRNAs were downregulated (Fig. 2b and Supplementary Table S1). The top 60 DEmiRNAs were presented in a heat map based on gene expression (Fig. 2e). A total of 1885 significantly DEmRNAs were identified by stringent thresholds (FDR< 0.05), among which 1116 DEmRNAs were upregulated, and 769 DEmRNAs were downregulated (Fig. 2c and Supplementary Table S1). The top 60 DEmRNAs were presented by a heat map based on gene expression (Fig. 2f).

### Regulatory ceRNA network (DEcircRNA-DEmiRNA-DEmRNA) of rainbow trout under heat stress

**Fig. 3:**
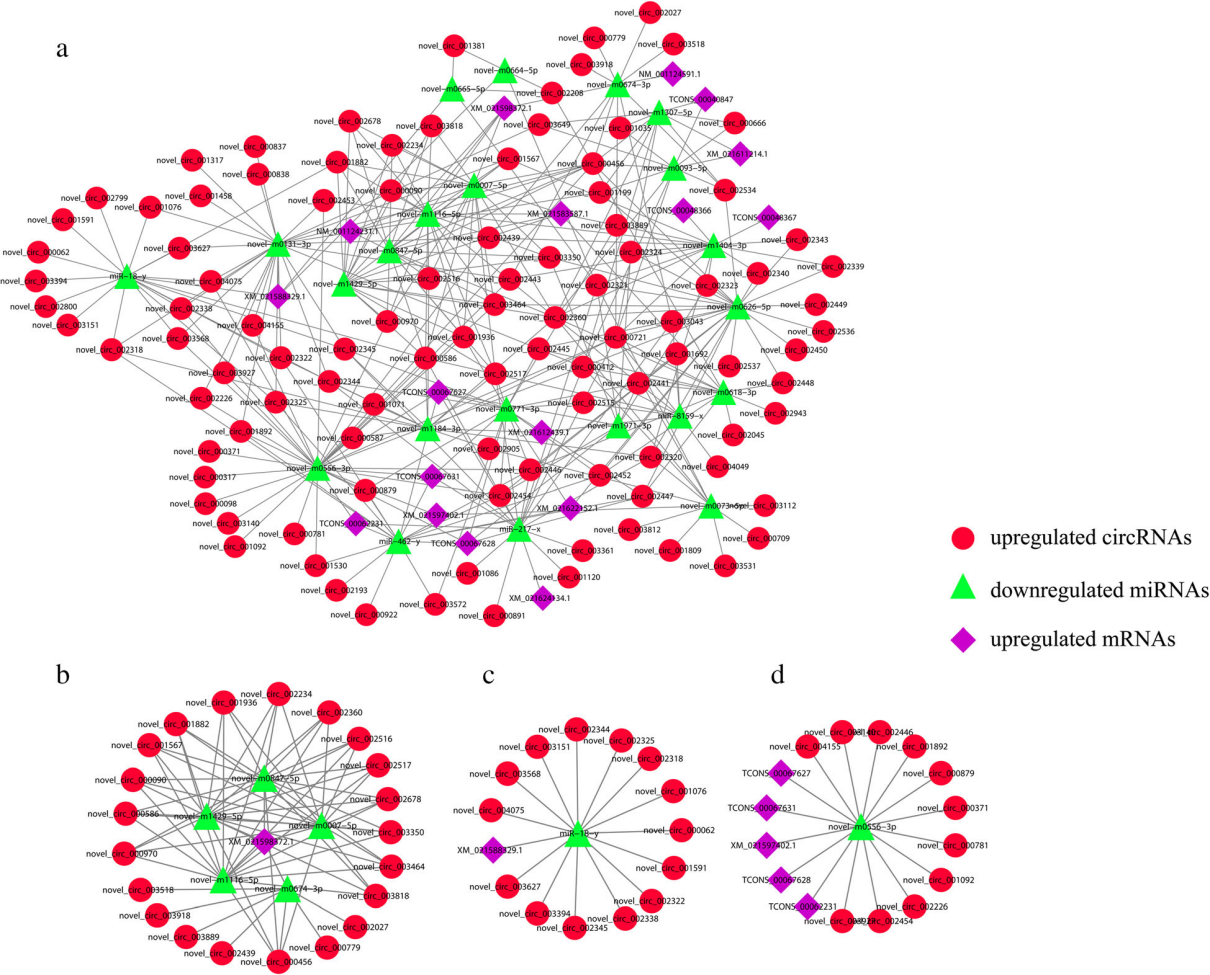
**a** ceRNA regulatory network in rainbow trout under heat stress. **b** Subnetwork of *novel_circ_003889 - novel-m0674-3p - hsp90ab1*. **c** Subnetwork of *novel_circ_002325 - miR-18-y - HSPA13*. **d** Subnetwork of *novel_circ_002446 - novel-m0556-3p - hsp70*

**Fig. 4:**
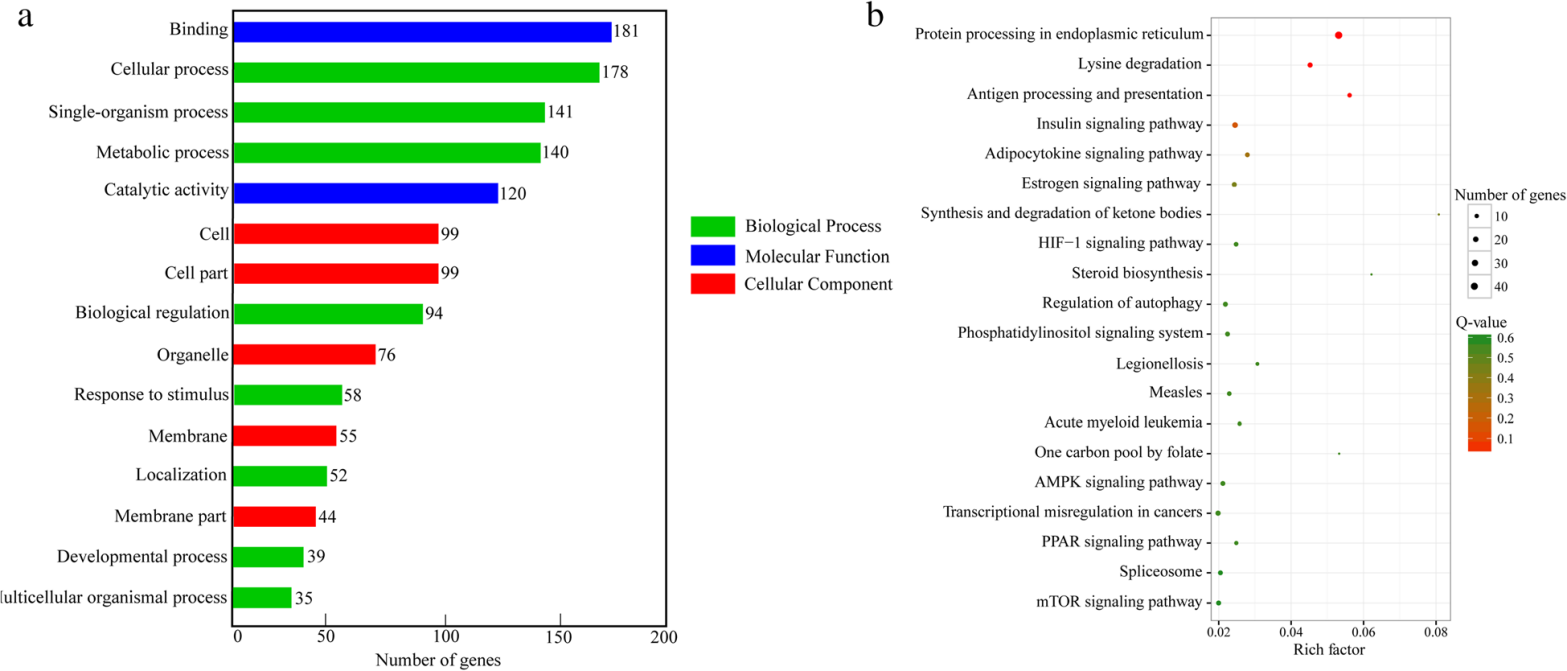
Top 15 GO terms (**a**) and top 20 KEGG pathways (**b**) in rainbow trout under heat stress

The ceRNA regulatory network contained 301 circRNA-miRNA pairs and 51 miRNA-mRNA pairs and included 103 circRNAs, 22 miRNAs, and 18 mRNAs (Fig. 3a and Supplementary Table S2). The subnetworks of XM_021598372.1 (*hsp90ab1*) were displayed in Fig. 3b, which shows that *hsp90ab1* expression was regulated by 21 circRNAs and 5 miRNAs. Among them, the most significant differences were in *novel-m0674-3p* and *novel_circ_003889*. The subnetworks of miR-18-y and novel-m0556-3p are displayed in Fig. 3c and d, respectively. The expression of *miR-18-y* was regulated by 14 circRNAs, which affect the expression of the target gene *HSPA13* (XM_021588329.1); among these circRNAs, the most significant difference was found in *novel_circ_002325*. Similarly, the expression of *novel-m0556-3p* was regulated by several circRNAs, but the expression of multiple target genes was affected. The most significant differences were found in *hsp70* (TCONS_00067628) and *novel_circ_002446*. Additionally, we performed Gene Ontology (GO) enrichment and Kyoto Encyclopedia of Genes and Genomes (KEGG) enrichment analysis on the DEmRNAs. GO enrichment analysis revealed that these DEmRNAs were significantly enriched in binding, single-organism process, metabolic process, catalytic activity, etc. (Fig. 4a and Supplementary Table S3). KEGG enrichment analysis showed that these target genes were significantly enriched in protein processing in the endoplasmic reticulum (ER), the estrogen signaling pathway, the Hypoxia-inducible factor 1 (HIF-1) signaling pathway, the PPAR signaling pathway, etc. (Fig. 4b and Supplementary Table S3).

### Validation of DEcircRNA, DEmiRNA and DEmRNA by RT-qPCR

**Fig. 5:**
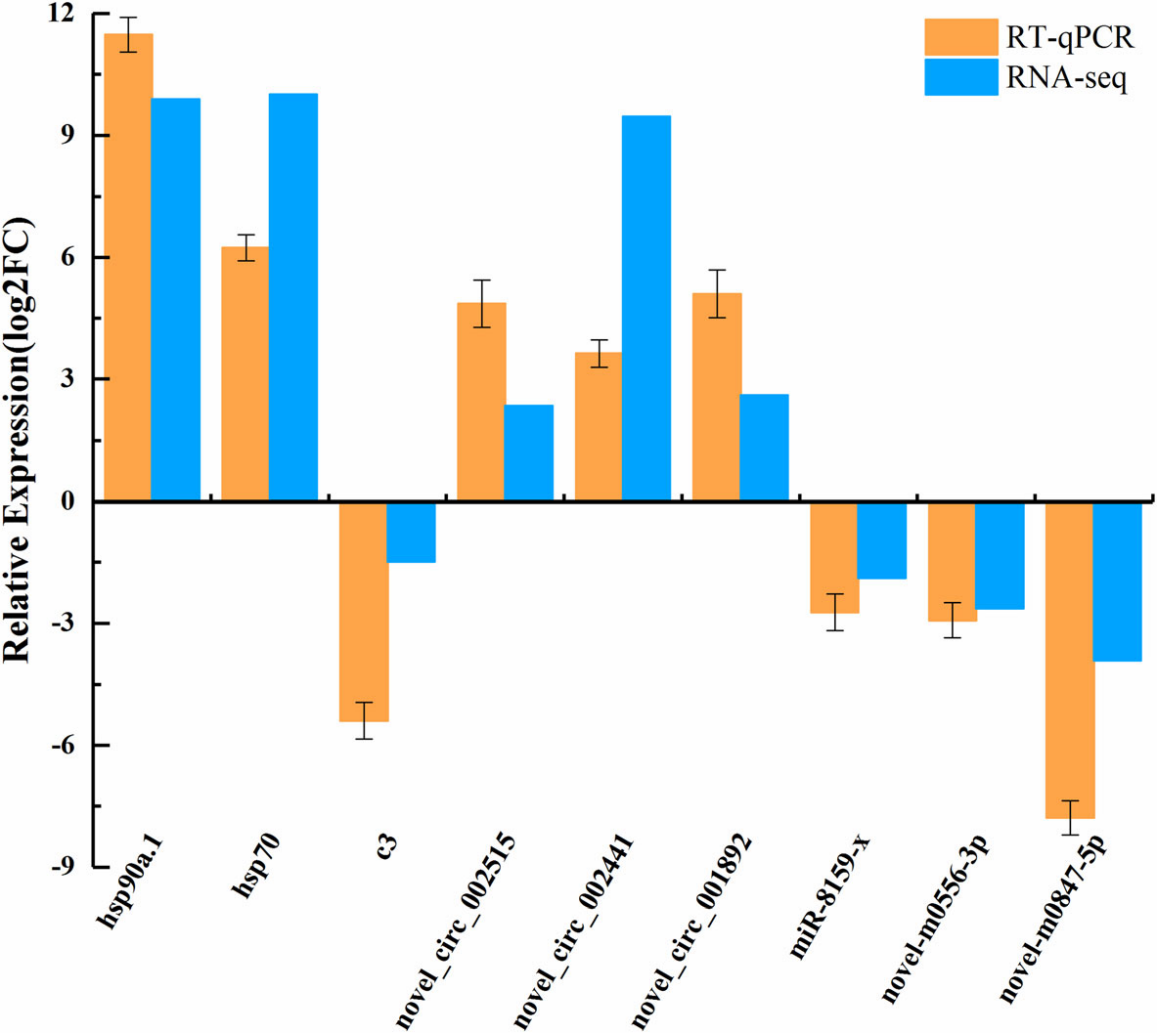
Comparison of the gene expression levels determined by RNA-seq and RT-qPCR

The expression levels of DEcircRNAs, DEmiRNAs and DEmRNAs were quantified in the CG and HS by using RT-qPCR. The relative expression (log2FC) of these DEGs was similar between the two approaches, although some quantitative differences were found between the RT-qPCR and RNA-seq analytical platforms (Fig. 5). Therefore, the RNA-seq results were reliable and can be used for bioinformatics analysis.

## Discussion

Stress response is a cellular response that is mainly caused by gene expression and regulation. When rainbow trout are subjected to heat stress, physiological functions are disturbed, intracellular protein denaturation and misfolding rates increased, and excess reactive oxygen species (ROS) are produced. Excessive ROS can lead to the production of metabolites such as MDA, which can cause extensive damage to nucleic acids, proteins and hyaluronic acid in cells, thereby destroying the oxidation-antioxidant system balance in cells and causing oxidative damage [4, 5, 27]. MDA content reflects the degree of ROS-induced damage in the organism. When the temperature increased to 24 °C, MDA accumulated in the rainbow trout, which indicates that cells are undergoing oxidative damage. We can infer that a large amount of ROS accumulated simultaneously and that a large amount of SOD was produced to remove the excess ROS. Therefore, SOD, ROS and MDA are dynamically regulated when the organism is subjected to thermal stimulation. An increase in LDH indicates that anaerobic glycolysis in cells becomes stronger under heat stress to ensure normal physiological activities and metabolism. Moreover, cell permeability is increased, leading to intracellular LDH escape; thus, the results indicate that the organism is undergoing stress or pathological changes [28, 29]. Alb and GLB are synthesized by the liver and participate in nonspecific immunity, such as repairing tissues and regulating plasma osmotic pressure. In the present study, GLB was significantly reduced, but Alb was significantly increased, revealing that liver cells were severely damaged under heat stress. In summary, changes in nonspecific immune parameters revealed that a strong stress response of rainbow trout was induced at 24 °C [5].

The organismal stress defense system is activated through the regulation of the expression of various RNAs, including both protein-coding and noncoding RNA. miRNAs bind to the MREs on target RNA transcripts, usually resulting in the repression of target gene expression [30, 31]. Specific circRNA may regulate various stress responses by inhibiting miRNA activity, and seminal studies of CDR1a revealed the important function of circRNA is as a sponge for miRNA [11, 32]. The comparison of the CG with the HS revealed that the quantity and expression level of DEcircRNAs, DEmiRNAs and DEmRNAs were significantly different in our study. Thus, the results suggested that rainbow trout may respond to heat stimulation by regulating the number and expression levels of various RNAs. Some important genes and noncoding RNA were identified under heat stress in the ceRNA regulatory network, such as *hsp90ab1*, *HSPA13*, *miR-18-y*, *novel-m0556-3p*, etc. We predicted three regulatory relationships: *novel_circ_003889* - *novel-m0674-3p* - *hsp90ab1*, *novel_circ_002325* - *miR-18-y* - *HSPA13* and *novel_circ_002446* - *novel-m0556-3p* - *hsp70* based on the ceRNA regulatory network and the most significant difference fold changes.

Heat shock protein (HSP) has been demonstrated to play crucial roles in protein folding, protein degradation, oxidation/reduction homeostasis, signal transduction, cellular response and innate immune function [33, 34]. Previous studies have shown that HSP90 plays important roles in increasing tolerance to the oxidative stress induced by water-borne heavy metals [35]. In our study, the most significant differences in expression levels under heat stress were observed for the HSP70 (e.g., *HSPA13*) and HSP90 (e.g., *hsp90ab1*) families; therefore, we speculate that these protein families were involved in important regulatory pathways in the organismal response to heat stress. Previous studies have shown that heat shock factor 2 (*HSF2)* were activated by the inhibition of *miR-18* in human cardiomyocytes [36]. Moreover, *miR-18* acts as a negative regulator that normalizes glucocorticoid receptors in mouse brains, thereby normalizing hypothalamic-pituitary-adrenal axis activity following stress exposure [37]. However, the activation of *HSPA13* is influenced by many circRNAs that act as sponges to inhibit *miR-18-y* expression in the ceRNA network, which may be due to the different purposes of miRNA regulation in different species or tissues. In addition, we also found that some miRNAs have multiple mRNA and circRNA targets (e.g., *novel-m0556-3p*).

When the biological defense system participates in the regulation of organismal stress, it will change the biological function of the organism; for instance, glucose metabolism is changed by cortisol secretion, etc. This change will lead to the development of a subpathological state and will inhibit immunity [38, 39]. We performed GO and KEGG enrichment analysis of DEGs in the ceRNA regulatory network under heat stress, and some important biological processes were enriched, such as metabolic process, biological regulation, response to stimulus, etc. We speculate that when rainbow trout were subjected to heat stress, the self-defense system responds to the stimulus through metabolism, hormone secretion, and other processes. Additionally, some important pathways were found, including protein processing in the ER, the estrogen signaling pathway, the HIF-1 signaling pathway and the PPAR signaling pathway, etc. Recently, research has indicated that the most significantly enriched KEGG pathway in the head kidney of rainbow trout in response to heat stress was ‘protein processing in the ER’, which is a quality control system that ensures correct protein folding or promotes the degradation of misfolded polypeptides by ER-associated degradation [6, 8]. The ER is a subcellular organelle in which proteins are folded with the help of luminal chaperones. Then, correctly folded proteins are packaged into transport vesicles that shuttle them to the Golgi complex, and misfolded proteins are retained within the ER lumen in complex with molecular chaperones. This pathway involves some HSPs, such as HSP40/70/90. Estrogen receptors are broadly expressed in many cell types involved in innate and adaptive immune responses and differentially regulate the production of cytokines [40]. Kang et al. [41] showed that under high temperature stress, many biological processes are extensively altered, particularly the estrogen signaling pathway, which is consistent with our results. Posttranslational histone modifications and miRNA and DNA methylation have been shown to influence the expression of ER-related genes and estrogen signaling genes [42]. Moreover, several coregulators of estrogen signaling also exhibit chromatin-modifying activities, further underlining the importance of epigenetic mechanisms in estrogen signaling. PPAR plays a role in the clearance of circulating or cellular lipids via the regulation of the expression of genes involved in lipid metabolism in the liver, lipid oxidation and cell proliferation [43]. HIF-1 is a TF that functions as a master regulator of oxygen homeostasis [44]. In this study, 12 DEGs were enriched in the HIF-1 signaling pathway, and we speculated that there may be two reasons for this result. First, the higher the temperature is, the lower the dissolved oxygen content is in the water, resulting in hypoxia stress in rainbow trout [45]. Second, HIF-1 is induced by other stimulants, such as heat stress, and causes changes in other metabolites such as NO or various growth factors [46].

## Conclusion

In conclusion, the present study enabled the systematic description of a ceRNA regulatory network and some biochemical parameters in rainbow trout under heat stress, and screened non-coding RNA in response to heat stress in rainbow trout. The numerous identified circRNAs, miRNAs and mRNAs provide references for further investigation into the regulatory mechanism of ceRNA. Our results also provide new insights into the molecular mechanisms of the heat stress response in rainbow trout that will be conducive to the development of strategies to prevent and treat high temperature stress-induced damage in cold-water fish. Meanwhile, for the screening of non-coding RNA also provides a reference thermal stress markers.

## Methods

### Animal and sample collection

Full-sib rainbow trout were purchased from a trout farm in Zhangye, Gansu Province, China. Sixty fish with a mean weight of 200 ± 5.5 g were transferred into a 6000 L aerated water tank and were cultured at 18 °C for 7 days. Prior to the experiment, the fish were randomly divided into two groups (30 per group) in 500 L water tanks and allowed to acclimate for another 7 days with a 12 h light/12 h dark photoperiod. During acclimate period, maintain micro-flow water and sufficient dissolved oxygen, feed normally (3 times/d), and the total daily feeding amount is 2% of body weight. To simulate temperature conditions in a natural environment, the water temperature in the HS was increased from 18 °C to 24 °C at a constant rate of 1 °C per 24 h using a temperature control system (Type: KDE-03A; Producer: Kedier). When the water temperature of the HS group reached 24 °C and maintained for 2 h, then samples were collected (no deaths occurred during adaptation and stress process). With the administration of a lethal dose (80.0 mg/L) of MS-222 (Sigma Aldrich Co., St. Louis, USA), the liver was harvested from twelve female fishes (eliminate the impact of gender differences and maintain the consistency of genetic background) from both the 18 °C CG and the 24 °C HS. Part of the tissue were used to measure biochemical parameters, and the remaining tissues were immediately flash frozen in liquid nitrogen and stored at − 80 °C for gene expression analysis.

### Measurement of nonspecific immunity parameters in the liver tissue of rainbow trout under heat stress

Twelve fresh samples from the CG (*n*=6) and HS (*n*=6) were used to determine superoxide dismutase (SOD) activity, lactate dehydrogenase (LDH) activity, albumin (Alb) content, globulin (GLB) content and malondialdehyde (MDA) content using commercially available kits purchased from Jiancheng Biological Project (Nanjing, China). Optical density was determined using an ELISA microplate reader (Thermo Scientific™ Varioskan™ LUX, FI-01620 Vantaa, Finland). Statistical analyse was performed using SPSS (version 19). The significance of differences in biochemical parameters was determined using Student’s t test, significance was set at *P*< 0.05, and all data were expressed as the Mean±SD.

### RNA extraction, strand-specific library construction and sequencing

Total RNA was extracted from the livers of twelve female fish from the CG (18 °C) and HS (24 °C), and the samples were equally mixed into six groups (e.g. the CG-A and CG-B were mixed in the same proportions, designated CG-1), namely, CG-1, CG-2, CG-3, HS-1, HS-2 and HS-3. After the total RNA was mixed, rRNAs were removed to retain mRNAs and ncRNAs. The enriched mRNAs and ncRNAs were fragmented into short fragments by using fragmentation buffer and reverse transcribed into cDNA with random primers. Second-strand cDNA was synthesized by DNA polymerase I, RNase H, dNTP (dUTP instead of dTTP) and buffer. Next, the cDNA fragments were purified with a QiaQuick PCR extraction kit and end repaired, poly(A) was added, and the fragments were ligated to Illumina sequencing adapters. Then, Uracil N-Glycosylase (UNC) was used to digest the second-strand cDNA. The digested products were size selected by agarose gel electrophoresis, PCR amplified, and sequenced using Illumina HiSeq™ 4000 by Gene Denovo Biotechnology Co. (Guangzhou, China).

### Identification of circRNA, miRNA and mRNA

The reconstruction of transcripts was carried out with the software Cufflinks, which, together with TopHat2, allows biologists to identify new genes and new splice variants of known genes. The program reference annotation-based transcripts (RABT) was preferred. We used the following parameters to identify reliable mRNA: (1) the length of the transcript was longer than 200 bp, and (2) the transcript contained more than 2 exons. Novel genes were then aligned to the Nr and KEGG databases to obtain protein functional annotation. Next, 20 mers from both ends of the unmapped reads were extracted and aligned to the reference genome to find unique anchor positions within the splice site. Anchor reads that aligned in the reversed orientation (head to tail) indicated circRNA splicing and then were subjected to find_circ to identify circRNAs. All of the clean tags were searched against the miRBase database to identify known miRNAs in rainbow trout. All of the unannotated tags were aligned with the reference genome according to their genome positions and hairpin structures predicted by Mireap_v0.2 software, and the novel miRNA candidates were identified.

### Differentially expressed circRNA, miRNA and mRNA in the liver tissue of rainbow trout under heat stress

To identify differentially expressed circRNAs, miRNAs and mRNAs between the CG and the HS, the edgeR package (http://www.rproject.org/) was used. We identified circRNAs and miRNAs with fold change ≥2 and *p*-value < 0.05 in a comparison between samples or groups as significantly DEcircRNA and DEmiRNA. Additionally, significantly DEmRNAs were identified with a fold change ≥2 and FDR < 0.05.

### Construction of the competing endogenous RNA (DEcircRNA–DEmiRNA–DEmRNA) regulatory network

The ceRNA network was constructed based on ceRNA theory as follows: (1) the expression correlation between mRNA-miRNA or circRNA-miRNA was evaluated using the Spearman Rank correlation coefficient (SCC). Pairs with SCC < − 0.7 were selected as negatively coexpressed circRNA-miRNA pairs or mRNA-miRNA pairs, and both mRNA and circRNA were miRNA target genes, and all RNAs were differentially expressed. (2) The expression correlation between circRNA-mRNA was evaluated using the Pearson correlation coefficient (PCC). Pairs with PCC > 0.9 were selected as coexpressed circRNA-mRNA pairs, and both mRNA and circRNA in these pairs were targeted and negatively coexpressed with a common miRNA. (3) A hypergeometric cumulative distribution function test was used to test whether the common miRNA sponges between the two genes were significant. As a result, only the gene pairs with a *p*-value less than 0.05 were selected.

### Quantitative real-time polymerase chain reaction (RT-qPCR) validation

RT-qPCR was performed on nine DEGs selected from RNA-seq data according to potential functional importance. Primers were designed according to transcriptome sequencing data of rainbow trout liver tissue using Primer Premier 5.0 (Supplementary Table S4). First-strand cDNA was synthesized using the Evo M-MLV RT Kit with gDNA Clean for qPCR (Accurate Biotechnology, Hunan, China). RT-qPCR was performed on each sample in triplicate using the SYBR Green Premix Pro Taq HS qPCR Kit (Accurate Biotechnology, Hunan, China) on a LightCycler®480 Instrument II (Roche, Switzerland) in a 20 μL reaction volume. *β-actin* was used as an internal control to normalize the gene expression level, as *β-actin* expression showed no change in response to temperature in preliminary experiments. The cycling parameters for the PCR amplification were as follows: 95 °C for 30 s, followed by 40 cycles at 95 °C for 5 s and 60 °C for 30 s. The amplification specificity was checked by melting curve analysis. The relative expression of target gene transcripts was calculated using the comparative Ct method (2^-ΔΔCt^) and subjected to statistical analysis with SPSS software (version 19).

## Supplementary Information

**Additional file 1: Table S1.** DEcircRNAs, DEmiRNAs and DEmRNAs in rainbow trout under heat stress.

**Additional file 2: Table S2.** ceRNA regulatory network in rainbow trout under heat stress.

**Additional file 3: Table S3.** GO enrichment and KEGG pathways in rainbow trout under heat stress.

**Additional file 4: Table S4.** RT-qPCR primers of circRNA, miRNA and mRNA in rainbow trout liver.

## Data Availability

All raw transcriptome data reported and sample metadata expression estimates in this article have been deposited to the National Center for Biotechnology Information (https://www.ncbi.nlm.nih.gov) under the accession number GSE135668 and GSE140112.
